# Characterization of Cholinergic System Dysregulation in the McGill‐R955‐hTau Rat Model of Human‐Like Tauopathy

**DOI:** 10.1002/alz70855_099587

**Published:** 2025-12-23

**Authors:** Quentin Bonomo, Sonia Do Carmo, Claudio A Cuello

**Affiliations:** ^1^ McGill University, Montreal, QC, Canada

## Abstract

**Background:**

Alzheimer's disease (AD), the most prevalent tauopathy, affects 6.7 million individuals aged 65 and older. While the retrograde atrophy of the basal forebrain cholinergic system is well‐documented in AD patients and amyloidosis‐based rodent models, the specific role of Tau pathology in cholinergic neurodegeneration remains poorly understood. This study utilized the McGill‐R955‐hTau rat model, which closely mirrors human‐like tauopathy, to investigate the extent to which brain tauopathy contributes to cholinergic system dysregulation in the absence of brain amyloidosis.

**Methods:**

Female and male 20‐month‐old homozygous transgenic and Wild‐type (WT) rats were utilized. At this time‐point, transgenic rats display Tau misfolding and aggregation into neurofibrillary tangle‐like inclusions leading to cognitive impairments, brain atrophy, neuronal degeneration and gliosis. To assess the density of cholinergic terminations, varicosities expressing the vesicular acetylcholine transporter (VAChT) were quantified in the parietal cortex via immunohistochemistry. Given that the phenotypic maintenance of cholinergic neurons is regulated by the endogenous nerve growth factor (NGF), we further investigated key proteins of the NGF metabolic pathway using Western Blot and Enzyme‐Linked Immunosorbent Assay.

**Results:**

Immunohistochemistry analyses point to a reduction in VAChT immunoreactive varicosities in McGill‐R955‐hTau transgenic rats compared to WT controls, indicating diminished cholinergic innervation in the cortex. Compared to WT littermates, transgenic rats also exhibited significantly reduced levels of mature NGF, underscoring a compromised trophic support necessary for the phenotypic maintenance of cholinergic neurons. In line with the above, proNGF and Neuroserpin levels were elevated in the Tau transgenic rats relative to WT controls, suggesting impaired conversion of proNGF into mature NGF.

**Conclusion:**

The McGill‐R955‐hTau rat model demonstrates cholinergic dysfunction at advanced stages of brain tauopathy, independent of amyloidosis. Our findings highlight a tau‐mediated cholinergic synaptic loss and an accelerated dysregulation of the NGF metabolic pathway previously linked only to amyloidosis or neuroinflammation. These findings offer a further insight into the mechanistic relationship between tauopathy and cholinergic system atrophy in the progression of AD pathology.

